# Effect of Thermal Treatment of Veneer on Formaldehyde Emission of Poplar Plywood

**DOI:** 10.3390/ma6020410

**Published:** 2013-01-30

**Authors:** Koji Murata, Yashuhiro Watanabe, Takato Nakano

**Affiliations:** 1 Division of Forest and Biomaterials Science, Graduate School of Agriculture, Kyoto University, Kitashirakawa oiwake-cho, Sakyo-ku, Kyoto 606-8502, Japan; E-Mail: tnakano@kais.kyoto-u.ac.jp; 2 Canon Inc., Ota-ku, Tokyo 146-8501, Japan; E-Mail: watanabe.yasuhiro548@canon.co.jp

**Keywords:** formaldehyde emission, poplar plywood, thermal treatment, Langmuir’s theory, Hailwood-Horrobin theory

## Abstract

A large amount of poplar plywood is now being imported into Japan from China, and as a result, formaldehyde emitted from this plywood represents an undesirable chemical that must be controlled using a chemical catching agent. The aim of this study is to find an approach to reduce the formaldehyde emission of poplar plywood using thermal treatment without employing any chemicals. The experimental results obtained show that heating veneer sheets in the temperature range of 150 °C to 170 °C effectively reduced the formaldehyde emission of plywood, without diminishing the mechanical properties of the veneer. By applying Langmuir’s theory and Hailwood-Horrobin theory to the adsorption isotherm obtained in this study, the relationship between the formaldehyde emission of plywood and the adsorption properties of veneer as a material is discussed. When veneer sheets were heated in the temperature range of 150 °C to 170 °C, the amount of hydrated water (monomolecular layer) decreased slightly and that of dissolved water (polymolecular layer) did not change. It is hypothesized that the formaldehyde emission of plywood is related to the condition of the adsorption site of the wood.

## 1. Introduction

Poplar is a fast growing tree that is planted in temperate regions, and in China, the industrial production of poplars has reached 50 million m^3^ per year [1]. Japan has recently begun to import a large volume of plywood from China, which accounts for up to a quarter of all its imported plywood [2]. Much of this plywood is made of poplar wood. Poplar has good quality, e.g., white color and ease of processing, and it has the potential to be used for many purposes. However, the use of poplar plywood remains limited in Japan (e.g., for packing material), on account of a number of problems it presents. One of these problems involves the difficulty of controlling the emission of formaldehyde [3]. Now, chemicals to control formaldehyde are being added to this plywood. If too much chemical solution is sprayed, the plywood may twist or warp, because of the change in moisture content. If a large amount of chemicals is added into an adhesive, the excess chemicals prevent the adhesive from being cured, and hence, the adhesive bonding of plywood may have insufficient strength. In addition, a new adhesive without formaldehyde would increase the present cost of the plywood [4,5,6]. The aim of this study is to produce poplar plywood using formaldehyde-type adhesives, but without the use of additional chemicals.

Formaldehyde is released from solid wood as well [7]. Because the quantity that is released from solid wood is less than that released from plywood, the emission of formaldehyde from poplar plywood may depend on a formaldehyde-type adhesive. Without a catalyst, formaldehyde combines with the hydroxyl groups in cellulose or hemicelluloses to form a hemiacetal [8]. Because this is a reversible reaction, a change in the equilibrium condition will cause a reverse reaction to release formaldehyde. It is assumed that the wood veneer may retain formaldehyde as a hemiacetal, which is formed by a chemical reaction between free-formaldehyde in the adhesive and cellulose/hemicellulose.

Poplar plywood tends to warp greatly with the adsorption of moisture [1]. This is also one of the problems presented by such plywood that may be caused by its high hygroscopicity. In this study, it is assumed that the formaldehyde emission of the plywood may be related to the hygroscopicity of the veneer. Bekahta *et al.* [9] explored the thermal treatment of veneer in order to improve its adhesive bonding. They reported that moderate heat treatment makes the surface of a veneer smoother and improves its bonding strength. It is known that heat treatment decreases the hygroscopicity of wood [10], a fact that may have a connection with the reversible reaction of formaldehyde stated above. Because excess heat treatment degrades the mechanical properties of wood, it is desirable that the veneer be heated at a moderate temperature, so the strength of the veneer does not decrease.

The aim of this study is twofold: (1) To find an approach to reducing the formaldehyde emission of poplar plywood using thermal treatment that does not employ chemicals and (2) to elucidate the mechanism of formaldehyde emission from such plywood.

## 2. Materials and Methods

### 2.1. Specimens 

Rotary-cut veneer sheets of Chinese poplar were imported. In China, poplar logs are transported to a veneer mill, and sheets of veneer are then distributed in the market. The sheet thickness was approximately 2 mm. The moisture content of the veneer was inconsistent, because the lathe veneer was seasoned outdoors in a Chinese mill. For this reason, the purchased veneer was dried using a hot press (at 130 °C) to 6% moisture content at the plywood works. In this test, the veneer that was dried using a hot press was imported from China. The prepared sheets of veneer were 400 mm × 400 mm in size and had a density of approximately 430 kg/m^3^. Hot press drying at 130 °C was taken into account, and the heat treatments were performed at four temperatures of 130 °C, 150 °C, 170 °C and 190 °C for 1 h. Three-ply plywood panels were manufactured out of preheated veneers using commercial melamine urea formaldehyde glue resin (Ohsika 640). Wheat flour as a filling material and urea were added to the resin. The glue was spread with a density of 180 g/m^2^, based on the wet mass, and was applied on one side of each veneer. The hot press pressure and temperature were 0.8 MPa and 125 °C, respectively, for 3 min of press time. The specimens of manufactured plywood were placed vertically on edge and were distant from one another to release formaldehyde from the surface. One sheet of plywood was prepared for each type of specimen.

### 2.2. Measurement of Formaldehyde Emission

The formaldehyde emissions were measured according to the Japanese industrial standard (JIS A 1460:2001) [11]. The formaldehyde emitted by the specimens of veneer and plywood were determined using the glass desiccator method (acetylacetone method). An estimation of the quantity of formaldehyde was obtained from the concentration of formaldehyde absorbed in 300 mL of distilled water inside the desiccator containing the specimens.

In comparison, the plywood specimens were made of Red meranti (*Shorea* sp.) and Chinese eucalyptus (hybrid *Eucalyptus grandis* × *E. urophylla*) that were produced in the same manner, and the formaldehyde emissions from both the veneer sheets and the plywood were measured.

### 2.3. Strength Test of Heated Veneer

The heated veneer sheets were cut into small specimens (65 mm (L) × 20 mm) and conditioned sufficiently in a climate chamber (20 °C, 65% of RH). A small specimen of veneers was loaded on the side that included lathe checks in a three-point bending test using a material testing machine (Shimadzu ADT-AV10k). In other words, the bending test was performed to place the tight side on the lower surface (tension part) and the loose side on the upper surface (compression part). The test span was 50 mm, and the loading rate was 5 mm/min. Fourteen small-specimens of each heat-temperature were prepared. The strength of the veneer sheets was evaluated in the bending test.

In terms of preparation, the weight loss brought about by heating the veneer at 150 °C and 170 °C for 1 h was 1.3% and 1.8%, respectively. In order to address the weight loss produced by heating in a temperature range from 100 °C up to as high as 150–170°C, the weight loss due to thermal decomposition was measured using thermogravimetry (TG, Shimadzu TGA-50). After a poplar specimen (10 mg) was dried at 100 °C for 3 h, it was heated in nitrogen gas at up to 160 °C in increments of 0.3 °C/min, after which the temperature was maintained at 160 °C for 30 min.

### 2.4. Adsorption Properties of Heated Veneer

Small specimens of the veneers (60 mm (L) × 17 mm) were dried with a vacuum dryer and then placed in the chamber, which included a saturated solution of mineral salts. When the moisture of the specimens reached a condition of equilibrium at 20°C, the equilibrium moisture content (EMC) for each value of relative humidity (RH) was obtained. The mineral salts used in this test were LiCl, CH_3_COOK, MgCl_2_, K_2_CO_3_, NaBr, SrCl_3_, BaCl_2_ and K_2_SO_4_, and the considered values of RH were 11%, 22%, 33%, 43%, 57%, 71%, 88% and 97%, respectively. The moisture adsorption isotherms of each veneer were determined by the obtained EMC, and their absorption properties were analyzed using Langmuir’s theory and the Hailwood-Horrobin theory. The Langmuir adsorption equation is as follows [12,13]:

(1) $$\theta =\frac{bp}{1+bp}$$

where *θ* is the surface coverage, *p* is the vapor pressure and *b* is Langmuir’s adsorption coefficient, which is exponentially related to the positive value of the energy of adsorption, *E*, as follows:

(2) $$b=K\exp\left(\frac{E}{RT}\right)$$

where *R* is the gas constant and *T* is the temperature. The pre-exponential factor, *K*, is equal to the ratio of the adsorption and desorption coefficients. When *m* and *m*_0_ are the moisture content (g/g) and saturation concentration (g/g), respectively, *θ* = *m*/*m*_0_. The fractional RH, *h*, is the vapor pressure, *p*, divided by the saturated vapor pressure, *p*_0_ = 23.38 hPa (20 °C). The Langmuir adsorption equation is transformed as follows [14]:

(3) $$\frac{h}{m}=\frac{1}{b^{\prime }m_{0}}+\frac{h}{m_{0}}$$

(4) $$b^{\prime }=bp_{0}$$

where *b'* is the modified adsorption constant. Because Langmuir’s theory, which explains that the sorption of a monolayer can be adopted for a low RH, the Langmuir adsorption coefficients were obtained by fitting the equation into the EMC at 11%, 22%, 33% and 44% of RH. Next, the equilibrium constant of dry wood adsorbing dissolved water is defined as *K*_1_, and the constant of the vapor cohering to dissolved water is defined as *K*_2_. The Hailwood-Horrobin adsorption equation is as follows [15,16,17]:

(5) $$\frac{h}{m}=-ah^{2}+bh+c$$

(6) $$K_{1}=1+\frac{b^{2}+b\sqrt{b^{2}+4ac}}{2ac}$$

(7) $$K_{2}=\frac{-b+\sqrt{b^{2}+4ac}}{2c}$$

(8) $$W=18\sqrt{b^{2}+4ac}$$

Because the Hailwood-Horrobin equation can be adopted for all humidity values, the absorption parameters were determined with the MCs at all of the eight relative humidity values used in this test.

## 3. Results and Discussion 

### 3.1. Formaldehyde Emission 

**Figure 1:**
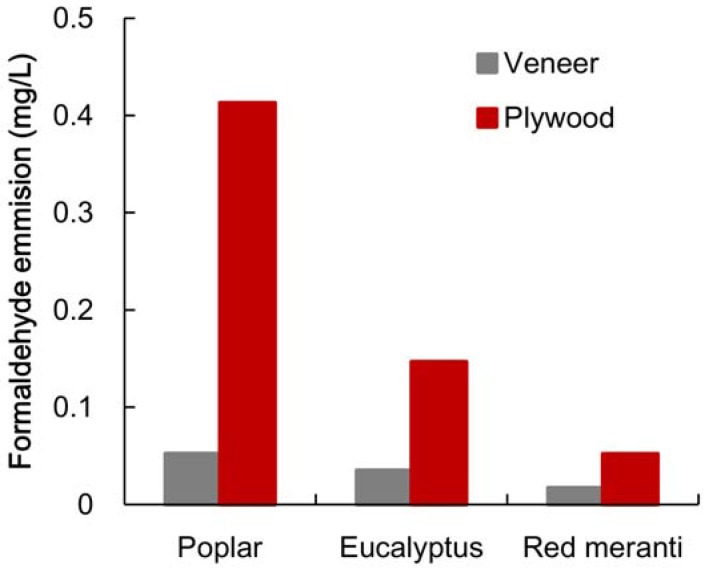
Formaldehyde emission of veneer and plywood.

The formaldehyde emission values for veneer sheets and for plywood without heat treatment for poplar, eucalyptus and red meranti are shown in Figure 1. The veneer sheets and plywood of poplar released more formaldehyde than those of eucalyptus and red meranti. There was, however, far less emission from the veneer sheets than from the plywood. The emission from the poplar plywood was 0.27 mg/L more than that from the eucalyptus. The difference, however, cannot be explained only in terms of the emission of the veneer sheets. It is understood that the large emission from poplar plywood is not caused by the release of the wood material itself.

**Figure 2:**
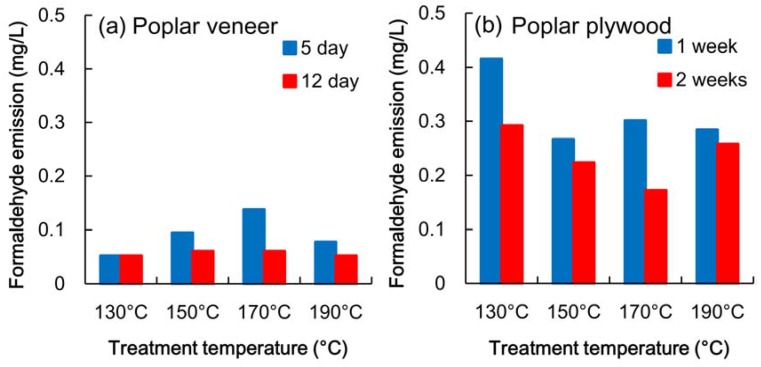
Formaldehyde emission of (**a**) heated poplar veneer and (**b**) plywood.

It has been reported that heat treatment facilitates the emission of formaldehyde from solid wood [18]. The formaldehyde emissions from the heated veneer sheets are shown in Figure 2a. Heat treatment at 130 °C did not increase the emission of formaldehyde, and treatment at higher than 150 °C facilitated the emission. After 12 days, the emission decreased to the same level as that of a veneer sheet that had not been treated. Lignin and hemicellulose have the potential for formaldehyde emission, and lignin seems to have a higher potential in this regard than cellulose and hemicellulose. Moreover, thermo-hydrolytical processing of wood can lead to formaldehyde emission from polysaccharides [19]. It was considered that formaldehyde produced by thermo-hydrolytical processing had been almost entirely released after 12 days (Figure 2a). In this test, the plywood was made of the veneer sheets that had been stored more than two weeks after heating.

The formaldehyde emission of poplar plywood made of heated veneer is shown in Figure 2b. The plywood treated at 130 °C released as much formaldehyde as did the poplar plywood that had not been treated, shown in Figure 1. When a veneer sheet was heated at a temperature higher than 150 °C, the formaldehyde emission of plywood decreased less than that at 130 °C. Moreover, this difference in emission level still remained after two weeks, and the emission of the plywood processed at 170 °C was the least of the emissions at all the study temperatures. These results indicate that the formaldehyde emission of plywood depends upon the interaction between the substance of wood and the formaldehyde in the adhesive.

A number of acid substances seem to be produced by the heating of veneers, and the pH of the veneer materials may thus be changed to some extent [20]. A strong acid condition will cause an irreversible reaction between formaldehyde and cellulose/hemicellulose to create cross-link hydroxyl groups. Moreover, melamine urea formaldehyde glue resin will harden rapidly under an acid condition. It is possible that the hardening of the resin can be accelerated by some acid substance that is produced by heating the veneer.

### 3.2. Mechanical Properties of Heated Veneer 

**Figure 3:**
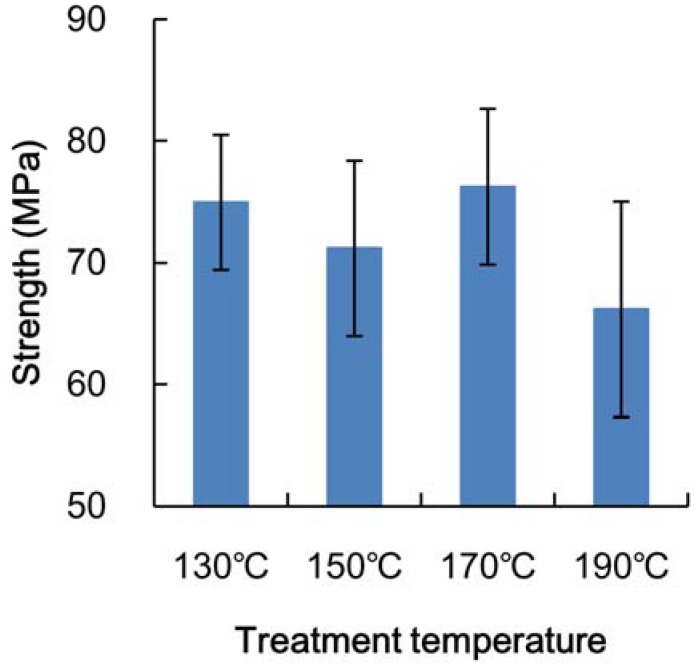
Strength of heated poplar veneer in bending test.

The strength of the heated veneer as obtained in a bending test is shown in Figure 3. The strength of the veneer did not decrease with heating at a temperature less than 170 °C, while at 190 °C, the strength decreased by more than 10%. When a *t*-test was performed to determine the statistical difference between heating at 130 °C, with a probability of a 1% level (degrees of freedom: 26), there was no statistical difference between the *t*-values of heating at 150 °C and at 170 °C (1.76 and 0.19, respectively). Heating at 190 °C, however, did produce a statistical significance (3.22). It was found that heat treatment at a temperature less than 170 °C had a small influence on the mechanical properties of the veneer, while heating at 190 °C could cause strong thermal decomposition.

**Figure 4:**
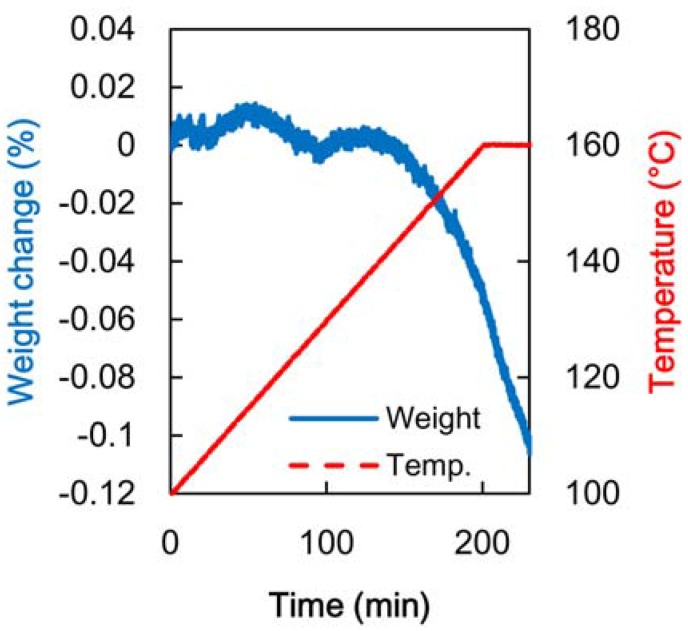
Weight loss determined by thermogravimetry (TG).

The weight loss obtained by TG is shown in Figure 4. It was found that a slight loss began at about 140 °C. The weight loss due to heating at 160 °C for 30 min was approximately 0.5%. Because the thermal degradation in the temperature range from 150 °C to 170 °C was very slight, the mechanical properties of the poplar veneer did not seem to be significantly influenced by this heat treatment. Shimizu *et al.* [21] studied the early stage of Xylan pyrolysis and reported that the weight average degree of polymerization hardly changed with heating at 160 °C up to 170 °C. It was considered that the pH of veneers hardly changed with heating at 160 °C up to 170 °C for 1 h, because of the very slight weight loss and non-degradation of the strength of the veneer sheets. Thus, it is thought that the acetal reaction to create cross-link hydroxyl groups hardly happened and that the hardening of the resin was not accelerated.

### 3.3. Adsorption Properties of Heated Veneer 

**Figure 5:**
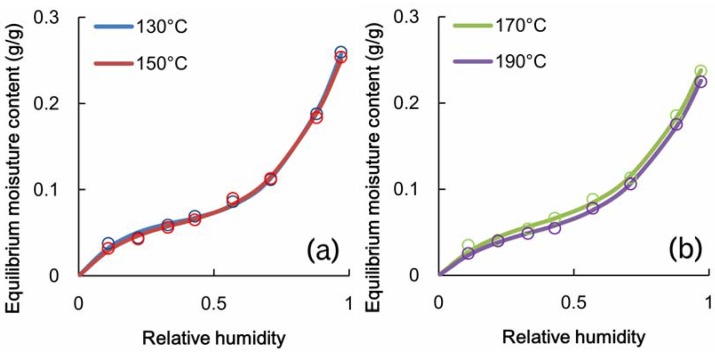
Adsorption isotherm of heated poplar veneer.

**Figure 6:**
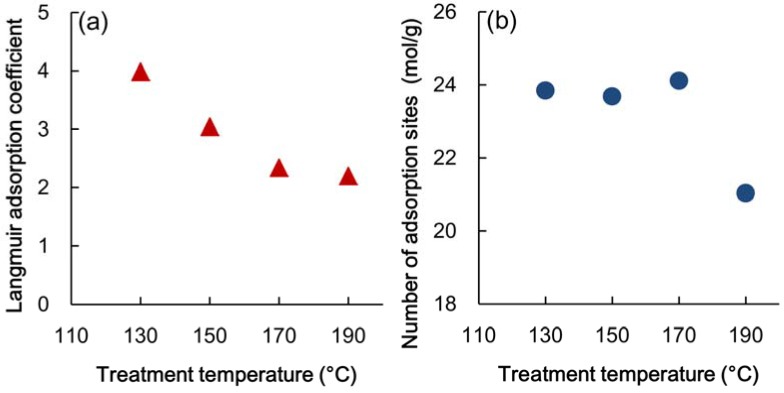
(**a**) Change in adsorption coefficient related to energy of adsorption; (**b**) adsorption sites per unit weight of wood substance.

The adsorption isotherms obtained in this test are shown in Figure 5. The lines represent the smoothing curves fitted to the resulting measurement values that were obtained using a cubic function though the origin. When the Langmuir equation was adopted for the isotherm, the Langmuir adsorption coefficients, *b'*, were as shown in Figure 6a. The adsorption coefficients tended to decrease with an increase in the heat temperature and then leveled off at 170 °C. The equilibrium constant of the Hailwood-Horrobin equation (*K*_1_*K*_2_) also displayed the same tendency as the Langmuir coefficients.

The change in the number of the adsorption sites per mole of wood substance (mol/g) is shown in Figure 6b. In the figure, we see that the number of adsorption sites did not change until 170 °C and that it abruptly decreased with heating at 190 °C. Kojiro *et al.* [22] reported that the volume of micropores decreased with heating at up to 160 °C in nitrogen gas and that in contrast, it increased with heating at a temperature higher than 190 °C. Shiga and Nakano [17] suggested that thermal degradation involves a decrease in the number of adsorption sites and gasification of the wood substance and that the surface properties of a wood substance depend upon the temperature at which it is heated. Thus, it is inferred that the heat treatment at up to 170 °C influenced only the adsorption sites on the surface, while the structure inside the wood substance did not change. Hydrated water (*M*_n_) and dissolved water (*M*_s_) were calculated using the following equation [10,16]:

(9) $$M_{n}\left(h\right)=\frac{18K_{1}K_{2}h}{W\left(1+K_{1}K_{2}\right)}$$

(10) $$M_{s}\left(h\right)=\frac{18K_{2}h}{W\left(1-K_{2}h\right)}$$

**Figure 7:**
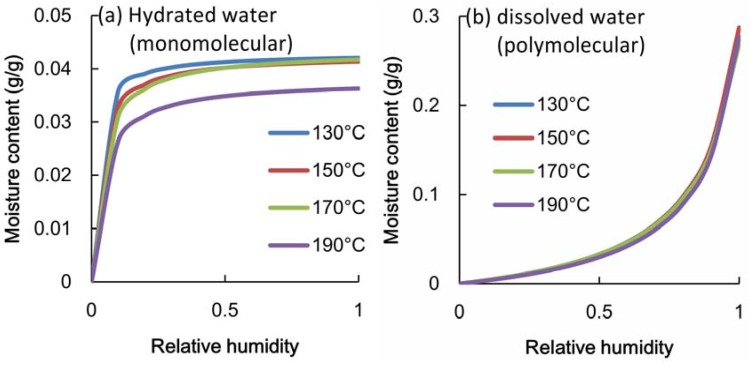
(**a**) Moisture content of hydrated water (monomolecular) in heated poplar veneer; (**b**) Moisture content of dissolved water (polymolecular) in heated poplar veneer.

The change in the hydrated water and dissolved water produced by heat treatment are shown in Figure 7. The volume of dissolved water decreased only with treatment at 190 °C and did not change at temperatures less than 170 °C. Dissolved water contributes to the swelling behavior of wood and is related to the internal structure of the cell wall. The volume of hydrated water, on the other hand, slightly decreased with treatment at 150 °C and 170 °C. An obvious difference between 130 °C and 150 °C in the values of RH from 0.1 to 0.3 can be found. These results show that heat treatment at 150 °C and 170 °C modified the adsorption properties of the monomolecular layer, but did not affect the internal structure of the cell wall.

## 4. Relationship between Adsorption Properties and Formaldehyde Emission 

When a veneer sheet was heated at a temperature higher than 150 °C, poplar plywood released less formaldehyde than in the case of a non-heated veneer. Heat treatment at 150 °C and 170 °C changed only the adsorption property of the monomolecular layer, but did not influence the internal structure of the cell wall. When plywood is glued with a formaldehyde-type adhesive, the formaldehyde released from an uncured adhesive may react with the hydroxyl groups of cellulose and hemicellulose to form a hemiacetal. Because the adsorption property of a monomolecular layer is related to the accessibility of the hydroxyl groups, it may have an influence on the hemiacetal reaction of the hydroxyl groups. The formaldehyde emission of plywood is probably caused by free formaldehyde that is produced by the adhesive reaction with the hydroxyl groups of the wood substance during the gluing process, and formaldehyde is re-released after the adhesive is cured.

Moreover, heat treatment at temperatures from 150 °C to 170 °C had a small influence on the strength of the wood, and the thermal degradation of plywood could be controlled if the veneer is heated in this temperature range. Thermal treatment at a high temperature is often used to improve the dimensional stability of wood, in a traded-off with its mechanical properties. If only formaldehyde emission is taken into consideration, it is interesting that the control of this emission is not a practical trade-off with the mechanical properties at a given temperature.

## 5. Conclusions 

To decrease the formaldehyde emission of poplar plywood, the effect of heat treatment upon the material of a veneer sheet was investigated. The results showed that when the veneer was heated at a temperature higher than 150 °C for 1 h, the emission from the poplar plywood decreased. Moreover, heating at temperatures less than 170 °C did not decrease the strength of the veneer sheet. The water adsorption properties of the heated veneer were estimated from the adsorption isotherms. When the poplar veneer was heated at 150 °C and 170 °C, the hydrated water (monomolecular adsorption) decreased slightly and the dissolved water (polymolecular adsorption) did not change. It is thought that formaldehyde emission is related to the adsorption properties of the monolayer and that free formaldehyde from uncured adhesive temporally reacted with the hydroxyl groups of the wood substance. Heat treatment at temperatures ranging from 150 °C to 170 °C was very useful, because such treatment can control the emission of formaldehyde from poplar plywood, while having little influence on its mechanical properties.
